# Photosynthetic Characteristics of Poplar–Soybean Intercropping Systems in Response to Phenolic Acid Stress

**DOI:** 10.3390/plants15091377

**Published:** 2026-04-30

**Authors:** Shuai Su, Chuanyu Zhang, Ning Chen, Liudong Zhang, Xingjian Dun, Xiaoyan Yu, Huilin Yang, Xia Wang, Tianyu Han, Changzhun Li, Hui Li

**Affiliations:** 1 College of Agriculture and Forestry Science, Linyi University, Linyi 276000, China; sushuai@lyu.edu.cn; 2 Shandong Academy of Forestry, Jinan 250014, China; chyuzhang@126.com (C.Z.); dxingjian@163.com (X.D.); wangxia99@126.com (X.W.); hantianyu4333@163.com (T.H.); lcz20150213@163.com (C.L.); 3 Forestry Station of Tanyi Town, Forestry Development Center of Fei County, Linyi 273411, China; dongbeiyuan123@163.com; 4 Shandong Provincial Forestry Protection and Development Service Center, Jinan 250014, China; zhangliudong@shandong.cn (L.Z.); 17662880205@163.com (X.Y.); yanghuilin@shandong.cn (H.Y.)

**Keywords:** photosynthesis, chlorophyll fluorescence, poplar–soybean intercropping system, biomass allocation, *Populus* × *euramericana* ‘Neva’, *Glycine max* (L.) Merr.

## Abstract

The continuous monoculture in *Populus* × *euramericana* ‘Neva’ plantations is closely related to the accumulation of phenolic acids in the soil, and these phenolic compounds exert a certain influence on plant nitrogen uptake. Leguminous plants can replenish soil nitrogen through biological nitrogen fixation, which is of great significance for enhancing plant productivity. This study employed different concentrations of phenolic acid treatments (0T, 0.5T, 1.0T, 1.5T, 2.0T) to analyze the photosynthetic characteristics of five phenolic compounds in a poplar–soybean (*Glycine max* (L.) Merr.) intercropping system, thereby providing a basis for biological management strategies aimed at increasing the yield of poplar monoculture stands. The results indicate that (1) *P*_n_ in poplar monoculture, soybean monoculture, and soybean intercropping all decreased as phenolic acid concentration increased, whereas *P*_n_ in poplar intercropping increased with rising phenolic acid concentration. Under treatments ranging from 0T to 1.5T, the decrease in *P*_n_ in the pure poplar, pure soybean, and intercropped soybean systems was primarily due to stomatal limitations, whereas under treatments ranging from 1.5T to 2.0T, it was primarily due to non-stomatal limitations. (2) Poplar, soybean, and soybean-intercropped poplar adapted to environmental stress by dissipating excess light energy absorbed by PS II as heat. The intercropping system effectively optimized poplar fluorescence parameters and mitigated the damage caused by phenolic acid stress to its photosynthetic machinery. (3) Chlorophyll A, chlorophyll B, and total chlorophyll in poplar and soybean leaves were significantly inhibited. (4) The biomass of poplars grown in monoculture decreased as phenolic acid concentration increased, whereas the biomass of poplars in intercropping showed the opposite trend. It is evident that, under phenolic acid conditions, poplar–soybean intercropping can mitigate the effects of phenolic acid stress to a certain extent.

## 1. Introduction

Poplar (*Populus* × *euramericana* ‘Neva’) is widely planted in northern China due to its rapid growth, strong resistance, and short rotation period. It is one of the primary tree species used in fast-growing, high-yield timber plantations in China and has played a significant role in meeting the country’s timber demand [1]. However, due to limited forest land resources, a management model involving continuous cropping of multi-generation stands is commonly adopted, leading to low productivity and the onset of continuous monoculture. Numerous studies have shown that the accumulation of phenolic compounds in the soil is one of the key factors contributing to poplar’s susceptibility to continuous cropping problems. Environmental stress can severely inhibit leaf photosynthetic efficiency, ultimately leading to reductions in plant growth, yield, and productivity [2]. Castaldi et al. [3] found that phenolic acids in the soil may alter microbial community composition and influence specific processes in the nitrogen cycle. Li et al. [4] found that phenolic compounds inhibit poplars’ uptake of ammonium and nitrate nitrogen, with the inhibitory effect increasing as phenolic concentrations rise. Castaldi et al. [5] found that soil phenolic compounds inhibit processes such as nitrogen fixation and transformation. Li et al. [6] demonstrated that the accumulation of phenolic compounds significantly inhibits soil nitrogen mineralization and nitrification, reducing the supply of available nitrogen in forest ecosystems and ultimately affecting tree growth. Xu et al. [7] noted that phenolic compounds alter the structure and composition of soil microbial communities, thereby regulating soil nitrogen cycling processes. Thus, phenolic acids may indirectly affect nitrogen uptake and utilization in poplar by modifying soil microbial processes.

Leguminous plants, with their biological nitrogen-fixing capability, can partially replenish soil nitrogen. When intercropped with non-leguminous crops, they significantly enhance the productivity of the latter. Zhang et al. [8] and Zhou et al. [9] have both conducted studies on the mulberry–soybean intercropping system, and their results consistently indicate that leguminous crops can lower soil pH and increase total soil nitrogen content; Zhang et al. [10] demonstrated that intercropping legumes can effectively modulate the arbuscular mycorrhizal fungal community and nitrogen-associated microorganisms in the soil, thereby promoting nitrogen utilization in intercropping systems. Similarly, Ren et al. [11] reported a gradual increase in the proportion of walnut nitrogen originating from legume nitrogen transfer. These studies demonstrate that legumes play a crucial role in improving soil microbial properties and enhancing nitrogen uptake and utilization by intercropped plants.

Soybean (*Glycine max* (L.) Merr.) is an annual herbaceous plant belonging to the genus Glycine in the Fabaceae family. It is an important food and cash crop that plays a vital role in China’s agricultural production. The nitrogenase complex reduces atmospheric nitrogen (N_2_) to ammonia (NH_3_), which is subsequently protonated and assimilated by plant tissues in the form of ammonium ions [12]. Previous studies have found that walnut-soybean intercropping significantly improves soil nutrient levels during the walnut growing season. This cropping system markedly increases soil organic matter, alkali-hydrolyzable nitrogen, and available phosphorus, which promotes the accumulation of soil nutrients, improves soil physicochemical properties, and enhances nutrient uptake and utilization by trees [13]; The walnut-soybean intercropping system effectively improves soil structural stability. At the same time, the organic carbon and nitrogen contents in soil aggregates of different particle sizes have increased, which helps improve soil nutrient status and promotes tree growth [14]; intercropping soybeans with apple trees can reduce soil moisture evaporation and increase fruit tree yields [15]. Currently, numerous scholars have conducted research on the interplanting of soybeans with various commercial tree species, focusing on improvements in nitrogen use efficiency, soil structure, and yield. However, the impact of interplanted soybeans on the productivity of the poplar–soybean intercropping system has not yet been reported.

Photosynthesis refers to the process by which green plants absorb light energy, assimilate carbon dioxide and water, convert solar energy into chemical energy to form organic compounds, and release oxygen in the process; it is the foundation of plant survival [16]. Numerous studies have shown that plant photosynthesis is closely related to the severity of stress. Mujahid et al. [17] pointed out that abiotic stress adversely affects the reproductive growth and photosynthesis of horticultural plants, and that various stress-mitigating agents can enhance plant tolerance to abiotic stress. Through their study of a maize–soybean intercropping system, Kou et al. [18] found that appropriate row spacing can improve the net photosynthetic rate of plants within the intercropping system. Zhang et al. [19] found through agroforestry studies that the *G*_s_, *T*_r_, and *C*_i_ values of apple trees in intercropping were higher than those in the monoculture treatment. In summary, photosynthetic physiology serves as a key indicator of crop growth status and stress tolerance. Both stress environments and cropping patterns significantly influence plant photosynthetic characteristics, and appropriate regulatory measures can improve photosynthetic performance. This provides an important reference for this study’s exploration of the photosynthetic response mechanisms in poplar–soybean intercropping systems under phenolic acid stress.

Therefore, this study used soybean (*Glycine max* (L.) Merr.) and the poplar clone ‘Neva’ (*Populus* × *euramericana* ‘Neva’) as research subjects to analyze the effects of phenolic acids on the photosynthetic characteristics of the poplar–soybean intercropping system. The findings provide a scientific basis for enhancing the productivity of continuously cropped poplar plantations and for evaluating the feasibility of soybean-based biological remediation of phenolic acid-contaminated soil.

## 2. Results

### 2.1. Effects of Phenolic Acid Concentration on Photosynthetic Gas Exchange in Poplar and Soybean

**Figure 1:**
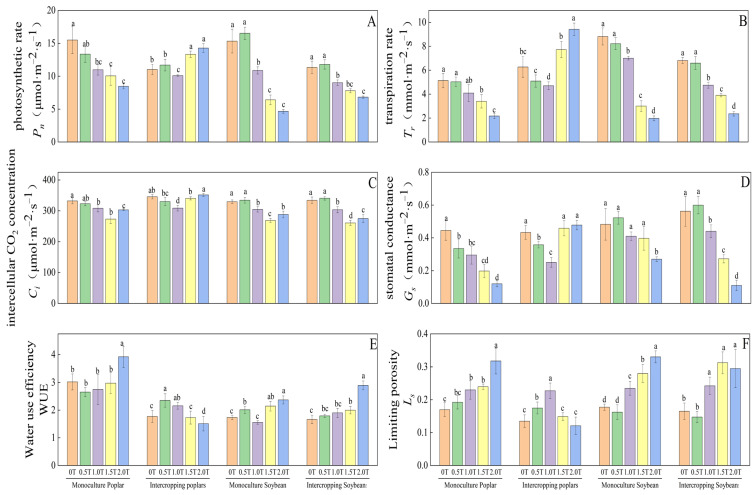
Effect of phenolic acid concentration on photosynthetic gas exchange in poplar and soybean at PAR = 1200 μmol·m^−2^·s^−1^. Note: Different lowercase letters indicate significant differences (*p* < 0.05) in phenolic acid concentrations among samples of the same plant. (**A**) photosynthetic rate; (**B**) transpiration rate; (**C**) intercellular CO_2_ concentration; (**D**) stomatal conductance; (**E**) Water use efficiency; (**F**) Limiting porosity.

As shown in Figure 1A, the net photosynthetic rate (*P*_n_) of monocultured poplar, monocultured soybean, and intercropped soybean all decreased with increasing phenolic acid concentration. In contrast, *P*_n_ in poplar intercropping systems showed an upward trend. Regarding cropping system effects, at phenolic acid concentrations of 0T, 0.5T, 1.0T, 1.5T, and 2.0T, the *P*_n_ of poplar intercropped with soybean was 71.11%, 87.59%, 92.07%, 132.62%, and 168.58% of that in pure poplar stands, respectively. As the concentration of phenolic acids increases, it is evident that intercropping with soybeans can, to some extent, enhance the photosynthetic productivity of poplars.

As shown in Figure 1B, the transpiration rate (*T*_r_) in monocultured poplar, monocultured soybean, and intercropped soybean decreased with increasing phenolic acid concentration. In contrast, *T*_r_ in intercropped poplar first decreased and then increased as phenolic acid concentration increased. At phenolic acid concentrations, the intercellular CO_2_ concentration (*C*_i_) significantly decreased in monocultured poplar, monocultured soybean, and intercropped soybean compared to the control treatment. However, between 1.5T and 2.0T, C_i_ increased as phenolic acid concentration rose (Figure 1C). For intercropped poplar, *C*_i_ decreased with increasing phenolic acid concentration below 1.0T but increased at concentrations above 1.0T. This suggests that *C*_i_ in the poplar–soybean intercropping system is influenced by both phenolic acid stress and intercropping effects.

As shown in Figure 1D,F, stomatal conductance (*G*_s_) in both monocultured poplar and monocultured soybean decreased with increasing phenolic acid concentration, while stomatal limitation (*L*_s_) increased. For poplar in the intercropping system, *G*_s_ first decreased and then increased with rising phenolic acid concentration, whereas *L*_s_ showed the opposite trend, first increasing and then decreasing. These opposing trends in *G*_s_ and *L*_s_ reflect the differential response of intercropped poplar to phenolic acid stress.

Based on the response patterns of the four photosynthetic gas exchange parameters—*P*_n_, *C*_i_, *G*_s_, and *L*_s_—to increasing phenolic acid concentration, and in accordance with the photosynthetic limitation criteria proposed by Farquhar and Sharkey [20], it is evident that under the 0T–1.5T treatments, the reduction in photosynthesis observed in pure poplar, pure soybean, and intercropped soybean was primarily due to stomatal factors. However, under the 1.5T–2.0T treatments, the decline in photosynthesis was constrained by non-stomatal factors. For poplar intercropped with soybean, stomatal factors remained the dominant constraint on photosynthesis across all phenolic acid concentrations (0T–2.0T), indicating that intercropping with soybean effectively mitigates the adverse effects of phenolic acids on the photosynthetic apparatus of poplar.

Regarding WUE, the highest values were observed in the leaves of poplar grown in pure stands, soybean grown in pure stands, and soybean grown in intercropped systems at a phenolic acid concentration of 2.0T, which was significantly higher than those under other phenolic acid treatments (*p* < 0.05). In contrast, the WUE of intercropped poplar exhibited a trend of initially increasing and then decreasing with rising phenolic acid concentration, peaking at a concentration of 0.5T. This change in WUE may be attributed to stress-induced stomatal closure rather than improvements in physiological performance.

### 2.2. Effects of Phenolic Acid Concentration on Fluorescence Parameters of Poplar and Soybean

**Figure 2:**
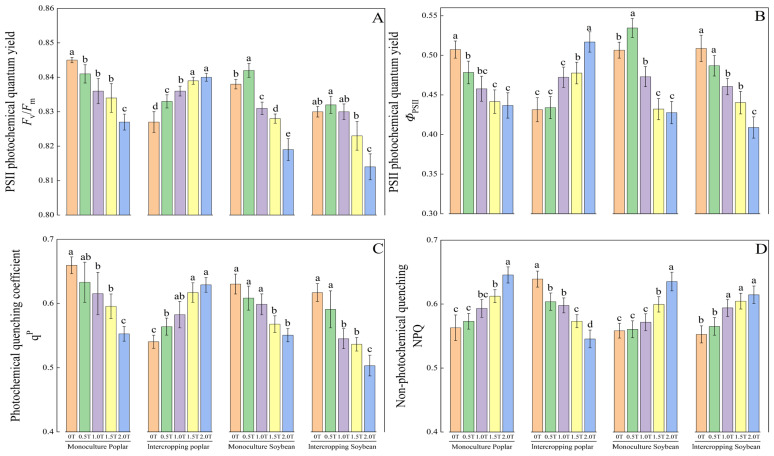
Effects of phenolic acid concentration on fluorescence parameters of poplar and soybean. Note: Different lowercase letters indicate significant differences (*p* < 0.05) in phenolic acid concentrations among samples of the same plant. (**A**) PSII photochemical quantum yield; (**B**) PSII photochemical quantum yield; (**C**) Photochemical quenching coefficient; (**D**) Non-photochemical quenching.

As shown in Figure 2A, at 2.0T, the *F*_v_/*F*_m_ of poplar in monoculture was significantly lower than that at other phenolic acid concentrations (*p* < 0.05). For both pure soybean and intercropped soybean, *F*_v_/*F*_m_ exhibited a trend of initially increasing and then decreasing with increasing phenolic acid concentration, reaching the highest values under the 0.5T treatment. For intercropped poplar leaves, *F*_v_/*F*_m_ increased with increasing phenolic acid concentration.

As shown in Figure 2B, *Φ*_PSII_ in pure poplar, pure soybean, and intercropped soybean all decreased as the phenolic acid concentration increased, whereas *Φ*_PSII_ in intercropped poplar increased with rising phenolic acid concentration. As the phenolic acid concentration increased, the qP of poplar in monoculture generally showed a decreasing trend (Figure 2C). For soybean in monoculture, there were no significant differences in qP among the 0T, 0.5T, and 1.0T phenolic acid treatments (*p* > 0.05), whereas the qP of intercropped soybean was significantly lower under the 2.0T phenolic acid treatment compared to other concentrations (*p* < 0.05). The authors speculate that this may be because the 2.0T concentration exceeds the growth threshold for self-regulation in intercropped soybean. Conversely, the qP of intercropped poplar increased with rising phenolic acid concentrations, with the 2.0T treatment yielding values of 116.43%, 111.56%, 108.00%, and 101.96% compared to the 0T, 0.5T, 1.0T, and 1.5T treatments, respectively. This suggests, to some extent, that intercropping can enhance the light energy utilization efficiency of poplar leaves under phenolic acid conditions.

As shown in Figure 2D, the NPQ of leaves from pure poplar, pure soybean, and intercropped soybean all increased with rising phenolic acid concentration, whereas the NPQ of leaves from intercropped poplar decreased with rising phenolic acid concentration.

### 2.3. Effect of Phenolic Acid Concentration on Chlorophyll Content in Poplar and Soybean Leaves

**Figure 3:**
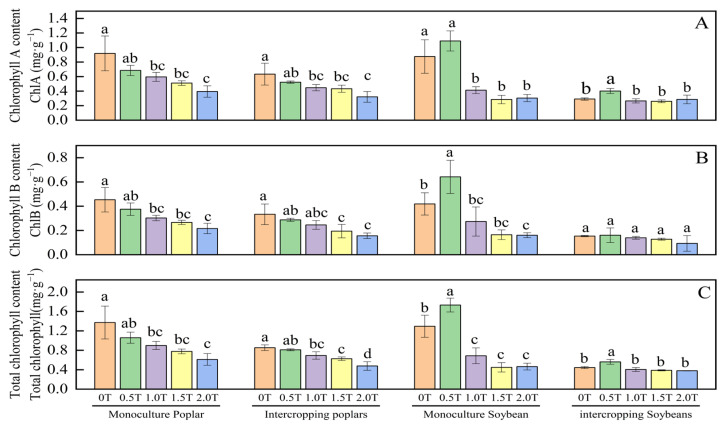
Effect of phenolic acid concentration on chlorophyll content (mg/g fresh weight) in poplar and soybean leaves. Note: Different lowercase letters indicate significant differences (*p* < 0.05) in phenolic acid concentrations among samples of the same plant. (**A**) Chlorophyll A content; (**B**) Chlorophyll B content; (**C**) Total chlorophyll content.

As shown in Figure 3A, the changes in ChlA content in poplar and soybean leaves did not follow the same pattern as phenolic acid concentration increased. In poplar monoculture, ChlA content in poplar leaves decreased as phenolic acid concentration increased; however, when intercropped with soybean, the ChlA content in poplar leaves under phenolic acid treatments of 0T, 0.5T, 1.0T, 1.5T, and 2.0T was 49.38%, 54.86%, 50.83%, 52.19%, and 54.76% of that in poplar monoculture, respectively. For soybean grown alone, the ChlA content in soybean leaves under the 1.0T, 1.5T, and 2.0T treatments was significantly lower than that under the 0T and 0.5T treatments. However, when intercropped with poplar, there was no significant difference (*p* > 0.05) in soybean leaf ChlA content among the various phenolic acid treatments, and all values were lower than those under the sole-cropping regime. This indicates that intercropping reduces the synthesis of ChlA in both soybean and poplar to some extent, which may be related to nutrient competition.

As shown in Figure 3B, the ChlB content in poplar and soybean leaves decreased as phenolic acid concentration increased. Specifically, for poplar leaves, the ChlB content consistently decreased with increasing phenolic acid concentration. In soybean leaves, ChlB content initially increased and then decreased as phenolic acid concentration rose, reaching its highest value under the 0.5T treatment. This indicates that soybean can promote ChlB accumulation at a phenolic acid concentration of 0.5T.

As shown in Figure 3C, total chlorophyll content in monocultured poplar decreased with increasing phenolic acid concentration, although no significant differences were observed among the 0.5T, 1.0T, and 1.5T treatments (*p* > 0.05). A similar trend was observed in intercropped poplar. Both monocultured and intercropped soybean reached their maximum total chlorophyll content under the 0.5T treatment, with values of 1.73 mg·g^−1^ and 0.56 mg·g^−1^, respectively. Overall, total chlorophyll content in monocultured poplar and soybean was significantly higher than that in their intercropped counterparts under the same phenolic acid treatment, with the difference becoming more pronounced at higher concentrations. In contrast, total chlorophyll content in intercropped poplar and soybean showed little variation with increasing phenolic acid concentration. This may be attributed to competition for nutrients and light within the intercropping system, although the underlying mechanisms require further investigation.

### 2.4. Effects of Phenolic Acid Concentration on Poplar and Soybean Biomass

**Figure 4:**
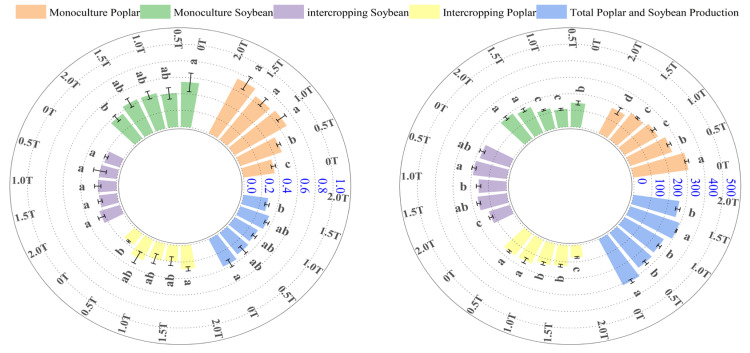
Effects of phenolic acid concentration on biomass and biomass allocation in poplar and soybean. Note: Different lowercase letters indicate significant differences (*p* < 0.05) in phenolic acid concentrations among samples of the same plant.

In terms of biomass allocation (Figure 4), the root-to-shoot ratio of pure-cultured poplar generally increased with rising phenolic acid concentration. Specifically, the root-to-shoot ratio under the 0T phenolic acid treatment was significantly lower than that under other phenolic acid stress conditions (*p* < 0.05). At phenolic acid concentrations of 1.0T, 1.5T, and 2.0T, there were no significant differences in the root-to-shoot ratio of pure poplar stands (*p* > 0.05). In the poplar-intercropped system, the root-to-shoot ratio decreased with increasing phenolic acid concentration; at concentrations of 0.5T, 1.0T, 1.5T, and 2.0T, it decreased by 34.28%, 55.34%, 47.57%, and 61.61%, respectively, compared to the poplar monoculture.

The root-to-shoot ratio of soybeans grown in pure stands showed little change with increasing phenolic acid concentration (*p* > 0.05), while that of soybeans in intercropped stands increased with rising phenolic acid concentration; the root-to-shoot ratio of the poplar–soybean intercropping system decreased with increasing phenolic acid concentration, which may be related to the intercropping system mitigating the inhibitory effect of phenolic acids on plant growth.

Overall, as phenolic acid concentration increased, a greater proportion of biomass in both pure-cultured poplar and soybean intercropping systems was allocated to the underground parts. Whether this is due to phenolic acids weakening root absorption capacity, thereby causing more biomass to be allocated underground, requires further investigation. Conversely, as phenolic acid concentration increased, more biomass from poplar in the intercropping system was allocated to the aboveground parts. The authors believe that this may be due to reduced damage to photosynthetic tissues and enhanced antioxidant capacity under the intercropping system. Overall, the intercropping system improves the poplar rhizosphere environment, alleviates phenolic acid stress, optimizes the supply of photosynthetic products, and promotes the allocation of biomass to the aboveground parts.

As shown in Figure 4, the biomass of poplar in a monoculture decreased as the concentration of phenolic acids increased. Specifically, the biomass under the 0T treatment was 125.02%, 142.30%, 156.27%, and 183.68% of that observed at phenolic acid concentrations of 0.5T, 1.0T, 1.5T, and 2.0T, respectively (*p* < 0.05). In contrast, the biomass of poplar in intercropping increased with rising phenolic acid concentration, contrary to the trend observed in poplar grown in monoculture. Specifically, under the 2.0T treatment, the biomass was 128.31%, 172.40%, 150.31%, and 110.67% of that observed at 0T, 0.5T, 1.0T, and 1.5T, respectively.

The biomass of soybeans grown in monoculture exhibited a trend of initially increasing and then decreasing with rising phenolic acid concentration, but it remained significantly higher than that of soybeans in the intercropping system under the same phenolic acid treatment. The total biomass of the intercropping system (poplar–soybean) exhibited a trend of first decreasing, then increasing, and finally decreasing again as phytic acid concentration increased.

## 3. Discussion

Photosynthesis is the core physiological process through which plants convert solar energy into chemical energy and synthesize organic compounds such as glucose. Approximately 90% to 95% of plant dry matter is derived from the accumulation of photosynthetic products, a process that plays a decisive regulatory role in crop organ differentiation, quality formation, and yield enhancement [21]. A decline in net photosynthetic rate (*P*_n_) is typically attributed to two categories of factors: stomatal limitation and non-stomatal limitation [22]. A study by Liu et al. [23] indicates that transcription factors are key regulatory elements in plants’ responses to abiotic stresses such as phenolic acids; they participate in defense responses to allelic stress by regulating antioxidant systems and hormonal signaling pathways. Under stress conditions, a significant reduction in stomatal conductance restricts CO_2_ diffusion into leaf tissues, leading to stomatal photosynthetic limitation. Non-stomatal limitation, in contrast, often results from damage to chloroplast structure, ultimately causing a marked reduction in photosynthetic physiological activity [24]. Wang et al.’s [25] research has shown that the reduction in photosynthesis in poplars under stress is primarily due to stomatal limitation.

In the present study, stomatal conductance (*G*_s_) in leaves of monocultured poplar, monocultured soybean, and soybean intercropped with poplar decreased with increasing phenolic acid concentration. Intercellular CO_2_ concentration (*C*_i_) also declined under 0T–1.5T treatments in these systems, accompanied by a decrease in *P*_n_ and an increase in stomatal limitation (*L*_s_), indicating that the photosynthetic reduction at this stage was primarily stomatal-limited. At phenolic acid concentrations of 1.5T–2.0T, photosynthesis in monocultured poplar, monocultured soybean, and intercropped soybean was mainly limited by non-stomatal factors. The decline in stomatal conductance and photosynthetic rate induced by phenolic acid stress may be closely related to abnormal modifications in cell wall composition and structure [26].

Li et al. [27] pointed out that under low-concentration phenolic acid stress conditions, the decline in poplar photosynthesis was primarily due to stomatal limitation, indicating that phenolic acids are a key factor limiting poplar photosynthesis. Similarly, the present study showed that at 0T–1.5T, increasing soil phenolic acid concentrations led to decreases in *P*_n_ and *C*_i_ and an increase in *L*_s_. According to the criteria of Farquhar and Sharkey [20], the primary cause of *P*_n_ reduction in monocultured poplar, monocultured soybean, and intercropped soybean was stomatal limitation (i.e., impaired CO_2_ supply to photosynthetic organs). Dou et al. [28] identified 1.5T as the critical concentration at which photosynthesis in poplar transitions from stomatal to non-stomatal limitation. However, the exact threshold concentrations for this transition in the three cropping systems examined here require further investigation. Under 1.5T–2.0T treatments, *C*_i_ increased significantly while *L*_s_ decreased markedly in monocultured poplar, monocultured soybean, and intercropped soybean, indicating that the subsequent decline in *P*_n_ was due to non-stomatal limitation, likely resulting from damage to the photosynthetic apparatus caused by high phenolic acid concentrations. Xie et al. [29] reported that with increasing phenolic acid concentration, *C*_i_ in poplar leaves rose significantly while *P*_n_ and *L*_s_ showed opposing trends, consistent with the present findings. The decline in photosynthesis caused by phenolic acid stress may be associated with disrupted nitrogen and iron uptake by the root system and nitrogen–iron imbalance in the plant [30].

This study also found that *P*_n_ in intercropped poplar was significantly higher under high-concentration phenolic acid stress than under low-concentration stress. The authors attribute this phenomenon to the potential nitrogen supply from intercropped soybeans via biological nitrogen fixation. Batool et al. [31] suggested that growth inhibition under stress may result from reduced photosynthetic rates, leading to impaired cell expansion and development. Elsalahy et al. [32] observed that mild stress slightly enhanced soybean leaf photosynthesis. However, in the present study, no significant differences in *P*_n_, *T*_r_, *C*_i_, *G*_s_, or *L*_s_ were detected between monocultured and intercropped soybean under 0T and 0.5T treatments, possibly due to differences in stress intensity or duration. Jo et al. [33] reported that photosynthetic rates in intercropped soybean were lower than those in monocultured soybean, raising the question of whether interspecific competition inhibits vegetative growth in intercropped soybean—a possibility that warrants further investigation.

Under normal conditions, light use efficiency (LUE) and water use efficiency (WUE) maintain a dynamic equilibrium [34]. However, under stress, the utilization of water and light energy within plants is altered. In this study, WUE increased with rising phenolic acid concentration in monocultured poplar, monocultured soybean, and intercropped soybean, whereas intercropped poplar exhibited the opposite trend. The authors propose that monocultured poplar and intercropped soybean preferentially allocate energy to water-related physiological processes. To avoid excessive water loss under elevated phenolic acid concentrations, they actively downregulate the transpiration rate (*T*_r_). Under phenolic acid stress, water absorption and transport are suppressed in monocultured poplar, monocultured soybean, and intercropped soybean, leading to disrupted water balance and ultimately reduced WUE. Numerous studies have demonstrated that damage to chloroplasts by reactive oxygen species impairs leaf photosynthesis. For instance, Wang et al. [35] observed irreversible damage in soybean plants under severe drought stress. Dubberstein et al. [36] investigated coffee plants under high-temperature stress and found that while most photosynthetic parameters recovered within 4–14 days after stress relief, some plants exhibited persistent aftereffects. Tu et al. [37] pointed out that phenolic acid stress disrupts the structure of the rhizosphere microbial community, thereby impairing nutrient uptake and causing an imbalance in photosynthesis. In the present study, photosynthetic activity in monocultured poplar, monocultured soybean, and intercropped soybean declined sharply under 1.5T–2.0T phenolic acid treatments, suggesting irreversible physiological damage.

Overall, intercropping with soybean can, to some extent, alleviate the inhibitory effect of phenolic acid stress on poplar photosynthesis and increase the photosynthetic productivity of intercropped poplar. The net photosynthetic rates of pure poplar, pure soybean, and intercropped soybean all decreased as phenolic acid concentration increased. Furthermore, between the 1.5T and 2.0T treatments, all three systems—pure poplar, pure soybean, and intercropped soybean—exhibited a threshold at which the photosynthetic rate shifted from being primarily controlled by stomatal factors to non-stomatal factors; the specific values require further investigation.

When photosynthesis is inhibited, photosystem II (PSII) in chloroplasts is the first to be affected. Elucidating the regulatory mechanisms of PSII under stress conditions can reveal plant adaptation strategies to adverse environments. Photosynthetic kinetics have become a focal point in plant physiology research, and related fluorescence parameters are widely used in studies of plant stress physiology [38]. Zhuang et al. [39] observed a decrease in *F*_v_/*F*_m_ in cucumber leaves under drought stress, while Zhang et al. [40] reported increased reduced *F*_v_/*F*_m_ in sorghum leaves under water stress. In the present study, as phenolic acid stress intensified, *F*_v_/*F*_m_ in monocultured poplar, monocultured soybean, and intercropped soybean all showed significant declining trends (Figure 2A), indicating that phenolic acid stress disrupts the structure and function of the PSII reaction center, thereby inhibiting photosynthetic physiological processes. In contrast, *F*_v_*/F*_m_ in intercropped poplar exhibited trends opposite to those in monocultured poplar, suggesting that intercropping effectively modulates the response of the poplar photosynthetic apparatus to phenolic acid stress.

With increasing phenolic acid concentration, the actual photosynthetic efficiency (*Φ*_PSII_) and photochemical quenching coefficient (qP) in monocultured poplar, monocultured soybean, and intercropped soybean decreased significantly, while the non-photochemical quenching coefficient (NPQ) increased markedly (Figure 2C,D). According to the principles of light energy absorption and allocation [41], under phenolic acid stress, the majority of absorbed light energy in these systems was dissipated as heat to mitigate stress damage and achieve self-protection. When photoinhibition occurs, plants typically exhibit reduced *Φ*_PSII_ and qP alongside increased NPQ. This aligns with findings by Yao et al. [42], who reported similar trends in Arabidopsis leaves under drought stress. In summary, the chlorophyll fluorescence parameters of monocultured poplar, monocultured soybean, and intercropped soybean all exhibited threshold responses, with significant changes occurring between 1.5T and 2.0T phenolic acid concentrations. In contrast, intercropped poplar showed increased *Φ*_PSII_ and qP and decreased NPQ under phenolic acid stress, possibly due to improved soil conditions and nutrient supply resulting from soybean biological nitrogen fixation and allelopathic effects, which regulate the functional state of the photosynthetic machinery.

In summary, as phenolic acid concentration increased, NPQ values for poplar monoculture, soybean monoculture, and soybean intercropping increased, while *F*_v_/*F*_m_, *Φ*_PSII_, and qP decreased. This indicates that phenolic acids caused damage to the PSII reaction centers in poplar monoculture, soybean monoculture, and soybean intercropping. In contrast, an increase in phenolic acid concentration in intercropped poplar resulted in a decrease in NPQ and an increase in *F*_v_/*F*_m_, *Φ*_PSII_, and qP, suggesting that the intercropping system enhances the light energy utilization efficiency of poplar under phenolic acid stress to some extent.

Chlorophyll pigments are key compounds involved in light capture and energy conversion in plants. Their content not only directly reflects photosynthetic capacity but also serves as an important indicator of plant physiological status [43]. Photosynthetic rate is positively correlated with total chlorophyll and chlorophyll A content. Chlorophyll A, as the central pigment in photosynthesis, enhances light energy absorption when its content increases [44]. Jo et al. [33] found that both chlorophyll A and total chlorophyll contents in intercropped soybean leaves were lower than in monocultured soybean. Geng et al. [45] reported significant suppression of chlorophyll A, chlorophyll B, and total chlorophyll in *Pinellia ternata* seedlings under salt stress. The present study revealed that phenolic acid stress significantly inhibited chlorophyll A, chlorophyll B, and total chlorophyll in both poplar and soybean leaves. Moreover, the chlorophyll content in intercropped poplar and soybean was lower than that in their monocultured counterparts, indicating that both phenolic acid stress and the cropping system (monoculture vs. intercropping) influence chlorophyll accumulation.

Biomass is a primary indicator of energy accumulation in plants and is influenced by plant size, environmental conditions, and developmental stage [46]. In this study, the biomass of individually planted poplar trees showed a decreasing trend as the concentration of phenolic acids increased. Nawaz et al. [47] demonstrated that biostimulants can promote soybean growth under stress conditions. In the present study, soybean biomass initially increased and then decreased with rising phenolic acid concentration, peaking at 0.5T. Whether this pattern reflects a mild stress-induced growth promotion in soybean requires further investigation. Zhang et al. [48] observed that a cotton–soybean intercropping system increased soil available nitrogen and phosphorus in the 0–20 cm layer. In this study, intercropped poplar biomass increased with rising phenolic acid concentration, whereas monocultured poplar biomass decreased, suggesting that soybean biological nitrogen fixation may meet nitrogen demands in the intercropping system.

Biomass allocation in plants always favors the direction of optimal growth, and the root-to-shoot ratio reflects how plants partition biomass between aboveground and belowground parts [49]. Li [50] reported that phenolic acid-treated poplar exhibited increased root-to-shoot ratios and altered biomass allocation patterns. In the present study, the root-to-shoot ratio of monocultured poplar and intercropped soybean increased with rising phenolic acid concentration, whereas that of intercropped poplar decreased. Root biomass in monocultured poplar increased under phenolic acid stress, indicating that intercropping alters the biomass allocation strategy of poplar, directing more biomass to aboveground tissues. In summary, phenolic acid stress modified biomass accumulation and allocation patterns in both poplar and soybean. Under monoculture, biomass was significantly suppressed by phenolic acids, and the increased root-to-shoot ratio reflected a passive adaptation to stress. Under intercropping, poplar biomass increased with rising phenolic acid concentration, likely due to effective nitrogen fixation by soybean, which alleviated phenolic acid stress and promoted aboveground biomass allocation.

Specifically, at a phytic acid concentration of 1.5T, the total biomass of the poplar–soybean intercropping system increased by 10.50% and 14.46% compared to the 0.5T and 1.0T treatments, respectively. In summary, phenolic acid stress had a significant inhibitory effect on the growth of poplar monoculture, soybean monoculture, and intercropped soybeans; the total biomass of the poplar–soybean intercropping system was highest under the 1.5T treatment.

## 4. Materials and Methods

### 4.1. Experimental Site Overview

The experimental site was located at the experimental field of Linyi University in Linyi City, Shandong Province (35°13′ N, 118°29′ E). This region features a temperate continental monsoon climate, with an average annual precipitation of approximately 900 mm and a mean annual temperature ranging from 12.6 °C to 13.9 °C. Soil type: Sandy loam soil. This study selected five major phenolic allelochemicals—p-hydroxybenzoic acid, ferulic acid, vanillin, benzoic acid, and cinnamic acid—from the poplar–soybean intercropping system. The test soil was collected from the experimental fields at Linyi University. The basic physicochemical properties of the soil are as follows: pH 6.58, organic matter content 11.30 g·kg^−1^, total phosphorus 0.70 g·kg^−1^, available phosphorus 28.01 mg·kg^−1^, total potassium 20.00 g·kg^−1^, and readily available potassium 111.70 mg·kg^−1^.

### 4.2. Experimental Seedlings and Treatments

This experiment utilized pot-grown plants and was conducted in a well-ventilated plastic greenhouse. During the experiment, light intensity inside the greenhouse reached 90% of outdoor levels, with temperatures ranging from 18 to 30 °C and relative humidity ranging from 41% to 65%. CO_2_ concentrations during the measurement period ranged from 380 to 400 μmol·mol^−1^. The plant materials consisted of one-year-old cuttings of poplar clone ‘Neva’ (*Populus* × *euramericana* ‘Neva’) and soybean seeds of the variety ‘Wansu 1208’. This planting method not only avoids competition from dense shade but also ensures an intercropped community structure, in line with the typical configuration logic of agroforestry systems.

In early April 2025, healthy, disease- and pest-free one-year-old seedlings in good condition were selected. After pruning, the cuttings should average 12 cm in length and 1.5 cm in diameter. Place the pruned cuttings in distilled water and change the water every two days. After approximately 2 weeks of cultivation, once root primordia are visible, plant the cuttings in pots filled with sterilized soil. To prevent phenolic acids from leaching out through the drainage holes at the bottom of the pots and reducing the concentration of phenolic acids in the soil, place a tray under each pot. In late April 2025, ‘Wansu 1208’ soybeans were selected as experimental material, with 8 plants planted in each pot. The pots used were 93 cm long, 32 cm wide, and 43 cm high, each containing 30 kg of soil. Prepare the treatment solution based on the phenolic acid content listed in Table 1, and apply 2 L of the diluted solution to the soil in each pot every two weeks. Keep the soil moist the rest of the time.

Three cropping systems were established: soybean monoculture, poplar monoculture, and poplar–soybean intercropping. Monoculture of poplar: 1 poplar tree; monoculture of soybeans: 8 soybean plants; poplar–soybean intercropping: 1 poplar tree and 8 soybean plants, in subsequent measurements, data were collected separately for the poplar intercrops and the soybean intercrops. For each system, different concentrations of phenolic acids were applied as shown in Table 1. Prepare phenolic acid solutions of varying concentrations, stir thoroughly to ensure uniform mixing, and let stand for a period of time to ensure even distribution of the phenolic acid. Apply phenolic acid once every 15 days. Each phenolic acid concentration treatment was set up with 8 pots as independent replicates, with each pot of plants serving as a single experimental unit.

### 4.3. Determination Methods

(1) Biomass Measurement

Collect all plants from each experimental plot. Poplar trees were harvested and their roots excavated. Each tree was then divided into four components: trunk, branches, leaves, and roots. Soybean plants were separated into aboveground and belowground parts. Each plant component was placed in labeled bags, oven-blanched at 105 °C for 30 min, and subsequently dried at 80 °C to a constant weight. Biomass was then weighed and converted to dry matter accumulation per plant [52].

The root–shoot ratio (RSR) was calculated as follows:

Root–shoot ratio (RSR) = Belowground biomass/Aboveground biomass

(2) Leaf Photosynthesis Measurement

In August 2025, photosynthetic characteristics were assessed. On clear mornings between 8:30 and 11:00, fully expanded, healthy leaves were selected from the upper canopy of the experimental plants. Select 8 pre-marked leaves from the upper and middle parts of plants in different treatment groups. The net photosynthetic rate (*P*_n_), stomatal conductance (*G*_s_), intercellular CO_2_ concentration (*C*_i_), and transpiration rate (*T*_r_) were measured on the 4th to 6th leaves from the apex using a Li-6800 portable photosynthesis system, with the light intensity set at 1200 μmol·m^−2^·s^−1^ [53]. The light intensity should be close to the light saturation point of the test crop to ensure that the leaves remain in a state of full light.

Other photosynthetic parameters were calculated using the following formula [54]:

Water use efficiency (WUE) = *P*_n_/*T*_r_

Light use efficiency (LUE) = *P*_n_/PAR

Limiting porosity (*L*_S_) = 1 − *C*_i_/*C*_a_

PAR: Photosynthetically Active Radiation; *C*_a_: Air Carbon Dioxide Concentration (*C_a_*)

(3) Measurement of chlorophyll fluorescence parameters

Chlorophyll fluorescence and photosynthetic gas exchange parameters were measured on the same morning. Select 8 pre-marked leaves from the upper and middle parts of plants in different treatment groups. Chlorophyll fluorescence parameters were determined using a pulse-modulated fluorescence meter (FMS-2). The measured parameters included initial fluorescence (*F*_o_), photosystem II (PSII), maximum fluorescence (*F*_m_), maximum fluorescence under light (*F*_m_’), steady-state fluorescence (*F*_s_), and minimum fluorescence under light (*F*_o_’) [55]. Before measuring the initial fluorescence (*F*_o_) and maximum fluorescence (*F*_m_), the leaves to be tested were dark-adapted for 30 min using leaf clamps, with a photosynthetically active irradiance of 1200 μmol·m^−2^·s^−1^. Additional chlorophyll fluorescence parameters were calculated using the following equations [56]:

PSII photochemical quantum yield (*F*_v_/*F*_m_) = (*F*_m_ − *F*_o_)/*F*_m_

Photochemical quenching coefficient (QP) = (*F*_m_’ − *F*_s_)/(*F*_m_’ − *F*_o_’)

Non-photochemical quenching (NPQ) = (*F*_m_ − *F*_m_’)/*F*_m_’

PSII photochemical quantum yield (*Φ*_PSII_) = (*F*_m_’ − *F*_s_)/*F*_m_’

(4) Chlorophyll Content Determination Method

Fresh leaves were collected and extracted using 80% acetone under low-temperature, light-protected conditions until the leaves had completely lost their color; chlorophyll content was determined according to the method described by Arnon [57]. Use 80% acetone (without plant material) as the blank control. Absorbance was recorded at wavelengths of 665 nm and 649 nm using a spectrophotometer (Shanghai McLean Biochemical Technology Co., Ltd., Shanghai, China). Chlorophyll concentration was calculated according to the following formula [58]:

Chlorophyll A (ChlA) = 13.95 × A665 − 6.88 × A649

Chlorophyll B (ChlB) = 24.96 × A665 − 7.32 × A649

Total chlorophyll (ChlA + ChlB) = ChlA content + ChlB content

Both chlorophyll A and chlorophyll B were measured at wavelengths of 665 nm and 649 nm. A665: Absorbance value at 665 nm wavelength; A649: Absorbance value at 649 nm wavelength.

### 4.4. Data Processing

Statistical analysis was performed using SPSS 16.0. First, the data were tested for normality and homogeneity of variances. Assuming normal distribution and homogeneity of variances, one-way ANOVA was used to compare differences among treatments. For multiple comparisons, Duncan’s multiple range test was employed to determine whether the differences were statistically significant at the 5% level of significance. The fluctuations in the data shown in the figure represent the standard error (SE). All figures in this paper were created using Origin 2021.

## 5. Conclusions

Intercropping with soybean can, to some extent, alleviate the inhibitory effect of phenolic acid stress on poplar photosynthesis and enhance the photosynthetic productivity of intercropped poplar. The net photosynthetic rate (*P*_n_) of poplar grown in monoculture, soybean grown in monoculture, and soybean grown in intercropping all showed a decreasing trend as soil phenolic acid concentration increased. Within the treatment range of 1.0 T–2.0 T, there exists a critical concentration beyond which the limiting factor for the decline in net photosynthetic rates of both poplar and soybean shifts from stomatal factors to non-stomatal factors. The specific value of this critical concentration requires further research to determine. In pure poplar, pure soybean, and intercropped soybean systems, the quantum yield of non-photochemical quenching (NPQ)—which regulates energy dissipation—dissipates excess light energy absorbed by PSII as heat, thereby protecting the PSII reaction center from damage. In contrast, intercropped poplar exhibits the opposite trend, effectively improving fluorescence parameters and mitigating the damage caused by phenolic acid stress to the poplar photosynthetic apparatus. The biomass of poplar grown in monoculture showed a continuous decline with increasing phenolic acid concentration, while low concentrations of phenolic acid exhibited a certain promoting effect on soybean growth; the trend in biomass of poplar grown in intercropping was exactly the opposite of that in monoculture. In summary, as the concentration of phenolic acid increased, the productivity of poplar in monoculture, soybean in monoculture, and soybean in intercropping decreased significantly, whereas the productivity of poplar in intercropping was markedly enhanced.

## Figures and Tables

**Table 1:** Soil phenolic acid concentration settings.

Soil Phenolic Acid Mass Concentration (μg·mL^−1^)	P-Hydroxybenzoic Acid	Vanillin Acid	Ferulic Acid	Phenylacetaldehyde Benzoic Acid	Cinnamic Acid
0T	0	0	0	0	0
0.5T	76	5.2	3.25	10.3	0.98
1.0T	152	10.4	6.5	20.6	1.95
1.5T	228	15.6	9.75	30.9	2.93
2.0T	304	20.8	13	41.2	3.9

Note: T in the table refers to the phenolic acid concentration in the rhizosphere soil of second-generation poplar plantations selected from Wang’s study [51].

## Data Availability

Data are contained within the article. Further inquiries can be directed to the corresponding author.
